# DNA G-quadruplexes are uniquely stable in the presence of denaturants and monovalent cations

**DOI:** 10.1016/j.bbrep.2022.101238

**Published:** 2022-02-26

**Authors:** Tanner G. Hoog, Matthew R. Pawlak, Benjamin F. Bachan, Aaron E. Engelhart

**Affiliations:** Department of Genetics, Cell Biology, and Development, University of Minnesota, 6-160 Jackson Hall, 321 Church Street SE, Minneapolis, MN, 55455, United States

**Keywords:** G quadruplex, DNA, Hofmeister, Denaturant, Nucleic acids

## Abstract

Ions in the Hofmeister series exhibit varied effects on biopolymers. Those classed as kosmotropes generally stabilize secondary structure, and those classed as chaotropes generally destabilize secondary structure. Here, we report that several anionic chaotropes exhibit unique effects on one DNA secondary structure - a G quadruplex. These chaotropes exhibit the expected behaviour (destabilization of secondary structure) in two other structural contexts: a DNA duplex and i-Motifs. Uniquely among secondary structures, we observe that G quadruplexes are comparatively insensitive to the presence of anionic chaotropes, but not other denaturants. Further, the presence of equimolar NaCl provided greater mitigation of the destabilization caused by other non-anionic denaturants. These results are consistent with the presence of monovalent cations providing an especially pronounced stabilizing effect to G quadruplexes when studied in denaturing solution conditions.

## Highlights

• G-quadruplexes exhibit the lowest sensitivity to denaturation by anionic chaotropes among several DNA secondary structures.
• In G-quadruplexes, the destabilizing effect of other denaturants is uniquely well-mitigated by the presence of sodium ions.
• This phenomenon affords a structure-specific means of modulating nucleic acid folding.

## 1 Introduction

The Hofmeister series is a classification system for small ions. This series ranks these ions in order of their propensity to exert diverse effects on solvents and their associated solutes, including modulation of the surface tension of solvent, of the solubility of biomolecules, as well as of stability of the secondary and tertiary structures of biomolecules [1]. Ions on one end of this series are described as chaotropes, and they generally decrease surface tension, increase protein solubility, and decrease the stability of folded states of biomolecules. Ions at the other end of the series are described as kosmotropes, and they exert opposite effects to those of chaotropes, increasing surface tension, decreasing protein solubility, and increasing the stability of folded states.

Nucleic acids represent an attractive platform by which to characterize the impact of solutes on secondary structure stability, since these molecules can form a range of regularly ordered secondary structures that exploit varied molecular recognition phenomena with unique folding characteristics. In this study, we have examined DNA sequences known to form several different secondary structures: a duplex, consisting of canonical Watson-Crick base pairs, G quadruplexes, consisting of Watson-Crick/Hoogsteen hydrogen-bonded quartets with a central channel of coordinated cations [2,3], and i-Motifs, consisting of hemiprotonated C–H^+^-C base pairs in an intercalated tetraplex structure. In this work, we studied several nucleic acid structures in the presence of a chaotropic ion (perchlorate), and we observed that G quadruplex structures are uniquely insensitive to perchlorate-induced denaturation. We further characterized G quadruplexes in the presence of several other chaotropes and a non-Hofmeister ion denaturant.

## 2 Materials and methods

DNA Oligonucleotides. The oligonucleotides length and sequences are found in Table 1.

**Table 1:** DNA sequences used in this work.

Name	Sequence	Length
Duplex40Top/Duplex40Bottom	GGT GTC AGT AAG CCA TTC GAG ATC CTC ATA GTC GTC TCA C GTG AGA CGA CTA TGA GGA TCT CGA ATG GCT TAC TGA CAC C	40 nt
HumTel	TTA GGG TTA GGG TTA GGG TTA GGG	24 nt
HumTel+Tetrad	TTA GGG GTT AGG GGT TAG GGG TTA GGG G	28 nt
HumTel-Tetrad	TTA GGT TAG GTT AGG TTA GG	20 nt
HumTel+LongLoop	TGG GTT AGG GAA TTC GGG TTA GGG	24 nt
TBA	GGT TGG TGT GGT TGG	15 nt
TBA+Tetrad	GGG TTG GGT GTG GGT TGG G	19 nt
TBA+2Tetrad	GGG GTT GGG GTG TGG GGT TGG GG	23 nt
AGRO100	GGT GGT GGT GGT TGT GGT GGT GGT GG	26 nt
cMyc22	CCC CAC CCT CCC CAC CCT CCC C	22 nt
iMotif19	CCC CTC CCC TCC CCT CCC C	19 nt
Py27	TTC CCC ACC CTC CCC ACC CTC CCC TAA	27 nt

### 2.1 UV–vis melting curves

A Cary 60 UV–Vis Spectrophotometer (Agilent) with an attached qCHANGER 6 Peltier temperature controller (Quantum Northwest), TC1/Multi Temperature Controller (Quantum Northwest), and EXT-440CU (Koolance) was used for all thermal denaturation melts. Samples were ramped from 15 °C to 95 °C and back twice, for a total of 4 melting curves (two heating and two cooling) at 1 °C increments. UV measurements were collected from 250 nm to 350 nm at 1 nm increments against a water blank.

### 2.2 Circular Dichroism Spectra

A JASCO J-815 Circular Dichroism Spectropolarimeter with an attached 6-sample Peltier Turret Cell Changer (Model MPTC-490S/15) was used for all CD spectra. Samples were blanked against samples minus the oligonucleotide of interest. Spectra were obtained with 1 nm increments and an average of 3 scans.

### 2.3 Sample preparation

DNA oligonucleotides were purchased from Integrated DNA Technologies (IDT) with standard desalting. Samples were prepared in water, lyophilized, and then resuspended in the desired salt concentration. This method allowed for saturated salt solutions to be used; all samples were prepared in this manner for consistency. Except where otherwise stated, final concentrations were 5 μM oligonucleotide (20 μM for CD spectra), 50 mM Li-HEPES pH 7.4, except for i-Motif sequences, for which sodium acetate/acetic acid pH 5.0 was used as buffer, and 1 mM disodium EDTA. 1 mm quartz cuvettes were used for all melts, filled to ca. 95% capacity, and capped with Teflon stoppers.

### 2.4 Data analysis

Of the acquired spectrum obtained for each temperature increment, a single wavelength was plotted against temperature to view the thermal denaturation curve of a sample. Melting curves for duplex were observed at A_260_, and G quadruplex and i-Motif melting curves were observed at A_295_.

NaSCN and NaI exhibit a high absorbance in the UV that overlaps that of DNA. The absorbance was too high at 260 nm to correct absorbance with a blank, so A_280_ was used, which gave clear thermal denaturation curves compared to A_260_. T_M_ of A_280_ matched those of A_260_ for the concentrations where a transition curve could be obtained (Supplementary Table 10).

T_M_s were determined by fitting a sigmoid to the melting transition using Igor Pro 8. Presented T_M_s are an average of all four melting traces. In some cases, a full sigmoid fit could not be fit due to too high or low of a transition. In these rare cases, the differential was used to confirm melting temperatures.

## 3 Results

**Fig. 1:**
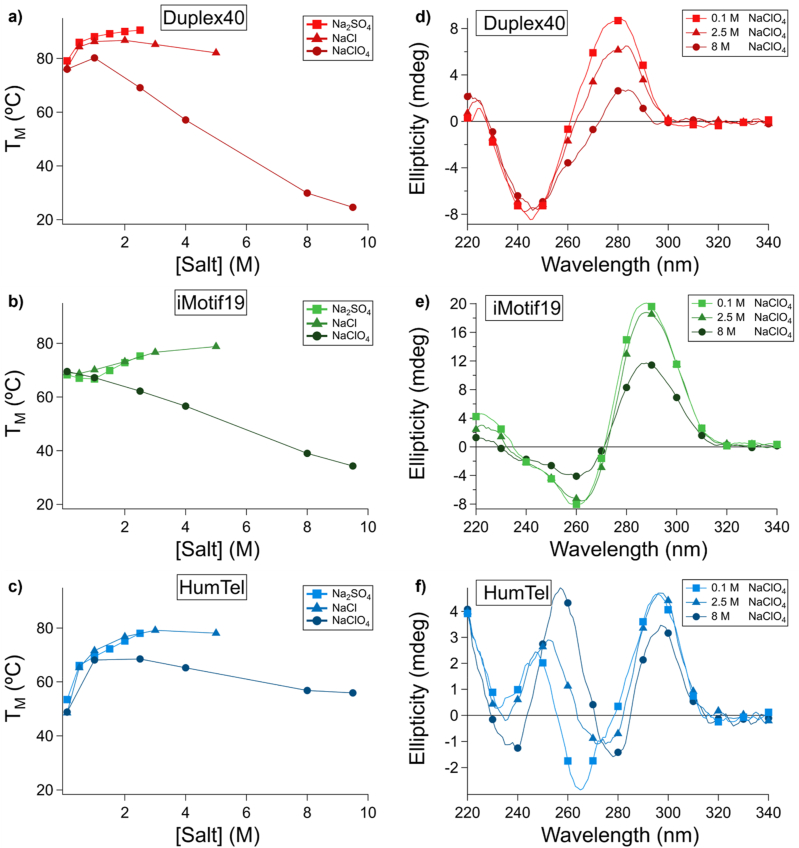
Melting temperatures of three representative DNA structures with varying [NaClO_4_]. a) The Watson-Crick duplex Duplex40 exhibits an increase in T_M_ between 0.1 and 1 M, presumably due to relief of electrostatic repulsion, whereas at higher concentrations of NaClO_4_, chaotropic effects predominate, with an average ΔT_M_/d[NaClO_4_] of −6.6 °C/M NaClO_4_ from 1 to 9.5 M NaClO_4_. b) The intramolecularly folded i-Motif iMotif19 does not exhibit an initial “electrostatic regime” of increasing T_M_, but it instead exhibits ca. linear dependence for T_M_ vs. NaClO_4_ intermediate to Duplex40 and HumTel, with an average ΔT_M_/d[NaClO_4_] of −3.9 °C/M NaClO_4_. c) The intramolecularly folded G quadruplex HumTel exhibits an initial increase in T_M_ as well (up to 2.5 M NaClO_4_), with a significantly smaller change in thermal stability at higher concentrations of NaClO_4_ and an average ΔT_M_/d[NaClO_4_] of ca. −2.1 °C/M NaClO_4_ from 2.5 M to 9.5 M NaClO_4_. d-f) CD spectra of Duplex40, iMotif19, and Humtel in varying [NaClO_4_].

We examined the thermal stability of a range of DNA structures (Table 1) in increasing concentrations of sodium perchlorate solution (Supplementary Table 1). A mixed-sequence duplex Duplex40 exhibited stabilization between 0.1 and 1 M sodium perchlorate, consistent with electrostatic effects predominating at low concentration and the increase in sodium ion concentration stabilizing the duplex. Above 1 M sodium perchlorate, duplex stability (as observed by the thermal midpoint monitored by UV absorbance) decreased monotonically with increasing perchlorate (−6.6 °C/M NaClO_4_, Fig. 1a). In contrast, the sodium salts of the kosmotropic ion sulfate and the Hofmeister-neural ion chloride had stabilizing and neutral effects, respectively (Fig. 1a). We next sought to investigate the impact of sodium perchlorate on other secondary structures. We examined a structure with a smaller secondary structure subunit and lower net charge - an unimolecular i-Motif (iMotif19) comprised of CH^+^-C base pairs [4]; this exhibited less destabilization with increasing perchlorate concentration (−3.9 °C/M NaClO_4_, Fig. 1b). Lastly, we assessed another four-stranded structure, a G quadruplex formed from the human telomere repeat (HumTel), which exhibited significantly less destabilization with increasing perchlorate concentrations (−2.1 °C/M NaClO_4_, Fig. 1c). Several i-Motif and G quadruplex structures we explored exhibited the same general trend (i-Motifs: −3.9 to −4.1 °C/M NaClO_4_, G quadruplexes: −2.1 to −3.3 °C/M NaClO_4_). (Supplementary Fig. 1, Supplementary Table 1).

These results were supported by circular dichroism (CD) spectra as well: duplex and i-Motif (Fig. 1d and e) structures exhibited spectral changes consistent with structure destabilization with increasing perchlorate, with Duplex40 exhibiting a decrease in the positive band at 280 nm, and iMotif19 exhibiting diminished spectral intensity consistent with a single-stranded deoxyribonucleotide oligomer. By contrast, HumTel exhibited attenuated destabilization and conformational switching with CD spectra consistent with a switch from antiparallel (characterized by positive bands at 295 nm and 245 nm and a negative band at 260 nm) topology to a hybrid (negative band at 245 nm and positive band at 264 nm) topology (Fig. 1f).

In order to measure perchlorate-specific effects separate from the influence of varying sodium concentration and ionic strength, we examined the stability of HumTel in perchlorate solution with constant 4 M sodium, using mixtures of sodium perchlorate and sodium chloride (Supplementary Fig. 2, Supplementary Table 2). Consistent with our observation that electrostatic effects predominate at lower salt, these high-salt solutions gave a linear decrease in stability with increasing perchlorate across the full concentration range (100 mM–4 M perchlorate) tested. The overall trend of enhanced sensitivity to perchlorate-induced destabilization for duplex vs. G-quadruplex remained consistent; Duplex40 exhibited a ΔT_M_/d[ClO_4_^−^] of −7.0 °C/M ClO_4_^−^, and HumTel exhibited a ΔT_M_/d[ClO_4_^−^] of −3.6 °C/M ClO_4_^−^, indicating an overall decreased sensitivity of G quadruplex to perchlorate-induced destabilization.

**Fig. 2:**
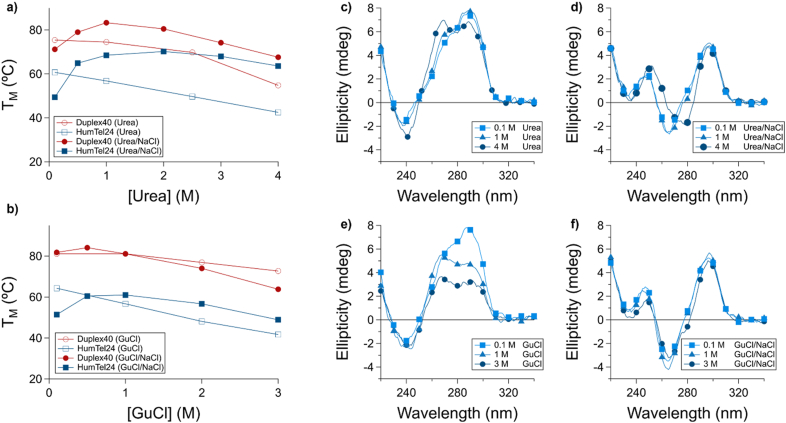
The inclusion of stoichiometric sodium chloride ameliorates the denaturing effect of urea and GuCl to a greater extent in G quadruplex versus Duplex DNA. a) The thermal stability of the duplex formed by Duplex40 (circles) and the G quadruplex formed by HumTel (squares) was measured in varying concentrations of urea with 100 mM KCl (open symbols) and equimolar urea/sodium chloride (closed symbols). Urea destabilized both structures (average dT_M_/d[Urea] of −6.6 °C/M from 1 to 4 M urea, Duplex40 and -4.7 °C/M, HumTel). The inclusion of equimolar sodium chloride at high denaturant concentrations decreased urea-induced destabilization to a greater extent in HumTel (dT_M_/d[Urea] = [NaCl] −1.7 °C/M from 1 to 4 M Urea/NaCl) than in Duplex40 (−5.3 °C/M from 1 to 4 M Urea/NaCl). b) A cationic chaotrope exerts similar destabilizing effects on a DNA duplex and G quadruplex. Duplex40 and HumTel both exhibit destabilization in the presence of increasing concentrations of 1–3 M guanidinium chloride (GuCl), with a greater destabilization for HumTel (dT_M_/d[GuCl]: HumTel, −7.5 °C/M; Duplex40, -4.2 °C/M). When equimolar sodium is added, HumTel is greatly stabilized relative to Duplex40 as measured from 1 to 3 M GuCl (dT_M_/d[GuCl] = [NaCl]: HumTel, −6.1 °C/M; Duplex40, -8.7 °C/M). c-f) CD spectra of HumTel in (c) Urea, (d) GuCl, (e) Equimolar Urea/NaCl, and (f) Equimolar GuCl/NaCl-containing solution.

We sought to examine the impact of the cationic component of sodium perchlorate independent of perchlorate-induced effects. To do so, we employed urea, a nonionic denaturant. We characterized the stability of the duplex secondary structure formed by Duplex40 and the G quadruplex formed by HumTel in two sets of conditions. In the first set of conditions, melting temperatures were measured in a fixed salt concentration (100 mM KCl) with varying concentrations of urea (Supplementary Table 3). Both secondary structures exhibited similar destabilization by urea under these conditions, with only slightly more urea-induced destabilization for Duplex40 (dT_M_/d[Urea]: HumTel, −4.7 °C/M; Duplex40, -6.6 °C/M; Fig. 2a). In a second set of conditions, melting temperatures were measured in solutions containing equimolar concentrations of urea and NaCl (Supplementary Table 4). NaCl was used to avoid Hofmeister-associated effects, since chloride is approximately neutral on the kosmotropicity-chaotropicity axis [5]. In these conditions, the denaturing effect of urea was mitigated to a greater extent for HumTel than for Duplex40 (dT_M_/d[Urea] = [NaCl]: HumTel, −1.7 °C/M; Duplex40, -5.3 °C/M; Fig. 2a). These findings agree with similar previously published results showing that increasing NaCl concentration can overcome urea-induced denaturation of G quadruplexes through central ion binding rather than electrostatic screening, as would be expected as the predominant means of cation-induced stabilization of DNA structures other than G quadruplexes [6].

We also examined the effect of varying concentrations of a cationic chaotrope, guanidinium chloride (GuCl), in the presence of a fixed concentration (100 mM) of KCl. We examined the stability of the duplex formed by Duplex40 and the G quadruplex formed by HumTel in these conditions (Supplementary Table 5). Both secondary structures exhibited similar destabilization by GuCl under these conditions, with only slightly greater destabilization for HumTel (dT_M_/d[GuCl]: HumTel, −7.5 °C/M; Duplex40, -4.2 °C/M; Fig. 2b). As a means of probing the influence of salt, we performed the same melts with equimolar GuCl and NaCl (Supplementary Table 6), and we found HumTel to exhibit attenuated destabilization relative to Duplex40 (dT_M_/d[GuCl] = [NaCl]: HumTel, −6.1 °C/M; Duplex40, -8.7 °C/; Fig. 2b).

We also examined the effects of urea and GuCl by circular dichroism. In the presence of potassium ion, HumTel exhibited a CD spectrum consistent with a hybrid conformation (positive band at 290 nm and shoulder at 265 nm). With increasing concentrations of urea, HumTel exhibited a spectral change consistent with a transition to a parallel G quadruplex (Fig. 2c, Supplementary Fig. 3a). When equimolar NaCl/urea mixtures were employed, the G quadruplex only existed in its antiparallel form and remained folded (Fig. 2d). This trend was also seen in the case of GuCl (Fig. 2e–f, Supplementary Fig. 3b).

The spectral changes consistent with a conformational change suggest the possibility of intermolecular G quadruplex formation [7], which may account for why HumTel is more stable relative to Duplex40 and iMotif19. We compared the melting temperature of HumTel in low (0.1 M) and high (8 M) sodium perchlorate and varied the amount of oligo in solution (1, 5, or 20 μM) and found no increase or decrease in melting temperature, ruling out the possibility of intermolecular complexes (Supplementary Table 7). We also examined a G quadruplex known to exist in parallel conformation, AGRO100 [8]. Like other G quadruplexes tested, it was resistant to perchlorate-induced denaturation (−2.1 °C/M NaClO_4_, Supplementary Fig. 4a). In sodium perchlorate, which was required due to the low solubility of potassium perchlorate, AGRO100 exhibited CD spectra consistent with a mixed conformation (Supplementary Fig. 4b) and concentration-dependent melting temperatures, consistent with intermolecular complexes (Supplementary Table 7). This is consistent with previous reports of this oligonucleotide [9,10].

**Fig. 3:**
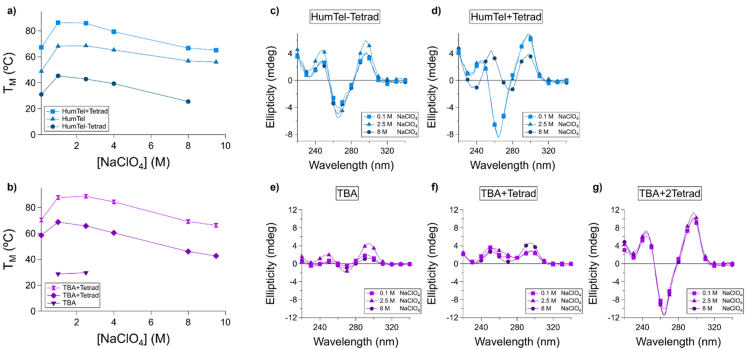
G quadruplexes of varied tetrad number exhibit the same destabilization trend in perchlorate solutions. a) A human telomere-derived G quadruplex (HumTel, 3 tetrads) as well as a more stable expanded analogue (HumTel + Tetrad, 4 tetrads) and less stable reduced analogue (HumTel-Tetrad, 2 tetrads) exhibit the same relative low sensitivity to perchlorate-induced destabilization. b) This is also observed for a second G quadruplex sequence (thrombin-binding aptamer, TBA, 2 tetrads) and two expanded (TBA + Tetrad, TBA+2Tetrad, 3 and 4 tetrads) analogues. c-d) In contrast to HumTel, which exhibits a conformational change with increasing sodium perchlorate (Fig. 1f) as observed by CD spectroscopy, (c) HumTel-Tetrad does not exhibit a large spectral change, (d) HumTel + Tetrad exhibit a structural change only at high sodium perchlorate concentrations, and (e–g) TBA and its derivatives do not exhibit large-scale CD spectral changes regardless of tetrad number.

G quadruplexes also exhibit increased stabilization with increasing numbers of stacked tetrads. This same trend is observed in perchlorate solutions with increased (HumTel+Tetrad) or decreased (HumTel-Tetrad) number of tetrads relative to HumTel (Fig. 3a). This trend was also observed for the thrombin binding aptamer G quadruplex, TBA (Fig. 3b) and its derivatives TBA+Tetrad and TBA+2Tetrad. We saw previously that as perchlorate concentration increased, HumTel exhibited a CD spectral change consistent with an antiparallel-to-parallel conformational change (Fig. 1f). This did not occur in HumTel-Tetrad, with one tetrad removed (Fig. 3c), but HumTel + Tetrad, with an additional tetrad, exhibited a spectral change consistent with this conformational shift (Fig. 3d). TBA did not exhibit spectral changes consistent with a conformational shift with increased tetrad number (Fig. 3e–g), consistent with the stability trends observed being due to tetrad stacking (*vs* conformational changes). These quadruplexes did not exhibit increasing T_M_ with increased oligonucleotide concentration, consistent with only intramolecular folding occurring (Supplementary Table 7).

**Fig. 4:**
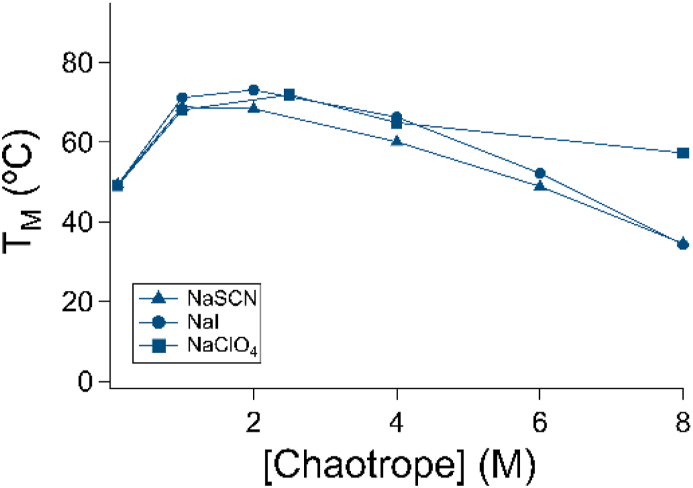
Sodium salts of multiple anionic chaotropes exhibit similar effects on a G quadruplex. HumTel exhibits similar behaviour with increasing chaotrope in the presence of NaSCN, NaI, and NaClO_4_.

Despite research on these ions dating back to the 19th century, the precise physical origins of Hofmeister ion-induced phenomena are not fully understood [11]. The origins by which these diverse ions exert their effects was long thought to be a result of disordering of water networks, but recent results have suggested ion-specific interactions are the predominant factor [1,12]. Thus, we sought to ascertain whether the effect we observed in sodium perchlorate-based systems was due to an ion-specific effect. To test this, we examined the stability of HumTel in the presence of varying concentrations of two other strongly chaotropic salts, sodium iodide and sodium thiocyanate (Fig. 4, Supplementary Tables 8–9). Sodium iodide and sodium thiocyanate exhibited similar effects to sodium perchlorate between 100 mM and 4 M. Thus, the behavior observed appears to be due to the cationic component of these salts, rather than due to Hofmeister anion-specific effects. Due to absorbance from the salts, complete CD spectra in NaI or NaSCN could not be obtained (Supplementary Fig. 5), but the region in which spectra could be obtained was similar to that observed in NaClO_4_ (Fig. 1f). Above 4 M, thiocyanate and iodine (−5.6 °C/M NaSCN, −6.5 °C/M NaI) exhibited slightly increased destabilization relative to NaClO_4_.

One loop-expanded analogue of the HumTel G quadruplex tested, HumTel+LongLoop, exhibited melting temperatures and CD spectra changes very similar to those observed for HumTel (Supplementary Fig. 6), consistent with quartet stacking driving the stability trends described here, but a systematic investigation of the impact of loop length is not reported here.

## 4 Discussion

We have observed that G quadruplexes are unique among DNA secondary structures in that the destabilizing effect of several denaturants is especially well-mitigated by the presence of monovalent cations. We have observed this is true of several denaturants, including a neutral denaturant (urea), a cationic chaotrope (guanidinium), and multiple anionic chaotropes. Thus, this effect appears to be general. One potential explanation for this phenomenon arises from the fact that G quadruplexes are distinct from other known secondary structure motifs, in that they coordinate a monovalent cation in their central channel by interaction with the O6 atoms on the guanine residues [13]. Thus, these structures are stabilized by cations in an additional mode beyond electrostatic screening [14]. Additionally, in very high concentrations of denaturant, water activity decreases. For example, a saturated solution of sodium perchlorate contains ca. 3 water molecules per ion. Accordingly, a decreased energetic penalty for cation dehydration could provide additional stabilization beyond that observed in bulk water [15]. Enhanced stability at lower water activity has also been observed for G quadruplexes in deep eutectic solvents, which are nearly anhydrous [16,17].

The lower degree of buried hydrophobic surface in DNA duplexes has been invoked as a reason for which Hofmeister salts favor protein folding, but not DNA folding [18]. Beyond the cation-specific effects we have observed here, the ca. 2 X larger buried surface (i.e., ca. 1 nm^2^ for a G-quartet vs. ca. 0.5 nm^2^ for a Watson-Crick base pair) is another reason G quadruplexes could exhibit anomalous responses to denaturants [19,20]. The varied effects of solute on DNA secondary structures could be exploited in the development of smart or switchable materials.

G quadruplex-duplex equilibria are of high interest in biology, given their presence in critical regions of the genome, such as promoters for proto-oncogenes, telomere DNA, and recombination hotspots [21]. DNA and RNA sequences that are capable of forming G quadruplexes can exist in equilibria with non-G quadruplex (i.e., duplex or single-stranded) states [22,23]. The precise suite of mechanisms by which biological systems modulate this equilibrium is not fully characterized. Several nucleic acid-binding proteins are known to alter G quadruplex folding, including ATP-dependent helicases [23], as well as non-helicase proteins, such as Lin28 and nucleolin, that do so in an energy-independent fashion [24,25]. Our observation of another means by which solutes exhibit differential effects on secondary structure stability suggests the enticing possibility that similar mechanisms could be operative in live cells, such as in phase-separated nucleic acid/protein domains [26].

## Author contributions

A.E.E. and T.G.H. designed research. All authors performed experiments and analysed data. A.E.E. and T.G.H. wrote the manuscript.

## Declaration of competing interest

There are no conflicts to declare.
