# Supplementary material for: DNA G-quadruplexes are uniquely stable in the presence of denaturants and monovalent cations
Source: Biochem Biophys Rep. 2022 Feb 26;30:101238. doi: 10.1016/j.bbrep.2022.101238 (PMC8885576; doi:10.1016/j.bbrep.2022.101238)
Supplement: Multimedia component 1 [file mmc1.docx]

Supplementary Information

For

**DNA G-Quadruplexes are Uniquely Stable in the Presence of Denaturants and Monovalent Cations**

**Supplementary Figure 1. Three i-Motif-containing DNAs exhibit similar sensitivity to sodium perchlorate denaturation.** None of the i-Motif-containing DNAs tested exhibited an “electrostatic regime” of increasing thermal stability with increasing NaClO_4_, and all exhibited similar average ΔT_M_/d[NaClO_4_]. Melting temperatures for **Py27** with 8 and 9.5 M perchlorate did not exhibit a full melting transition.


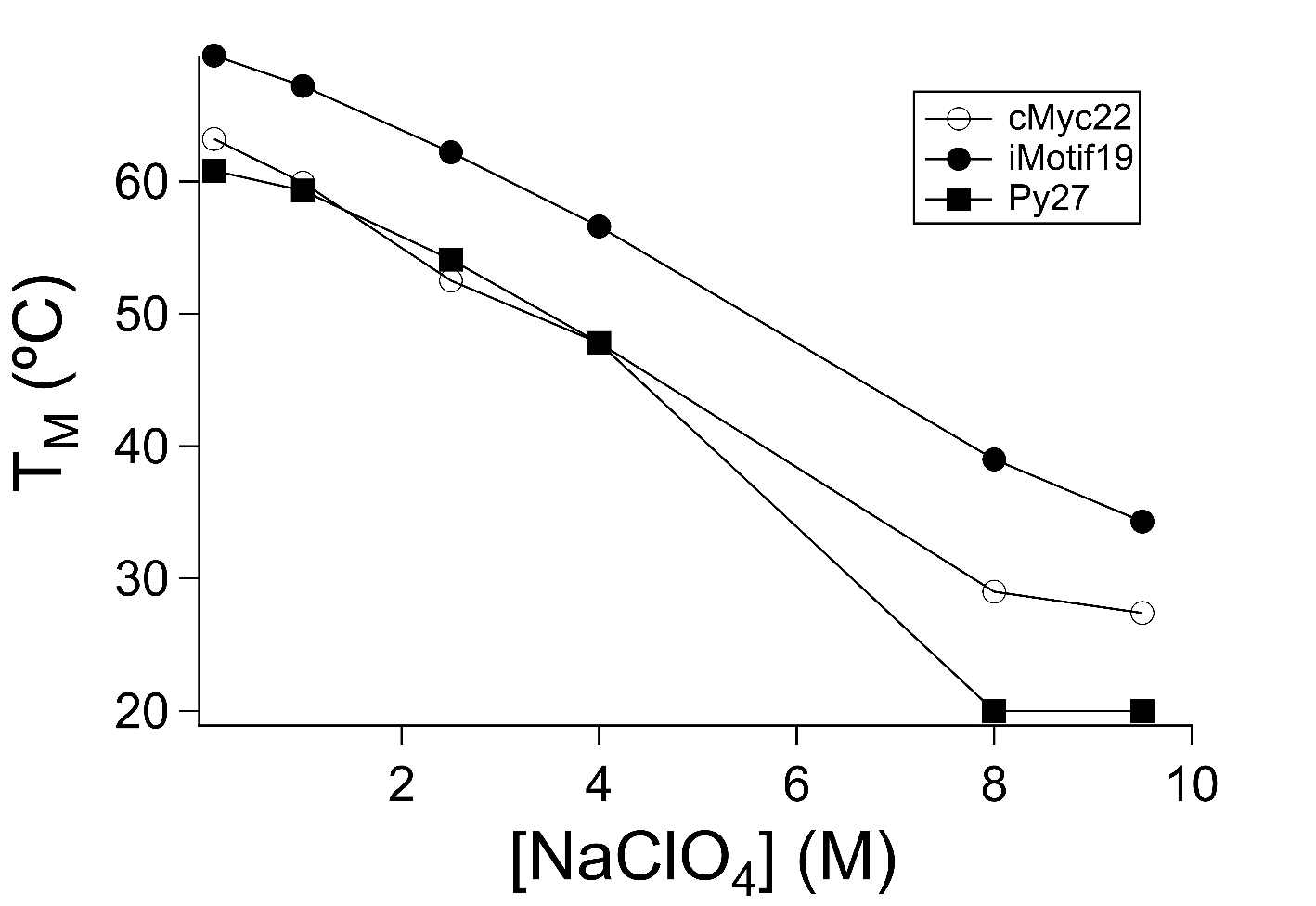


**Supplementary Figure 2.** (a) **HumTel** melting temperature vs perchlorate, with and without constant sodium provided by sodium chloride. CD spectra of **HumTel** in (b) constant 4 M sodium with varying perchlorate and (c) sodium perchlorate.
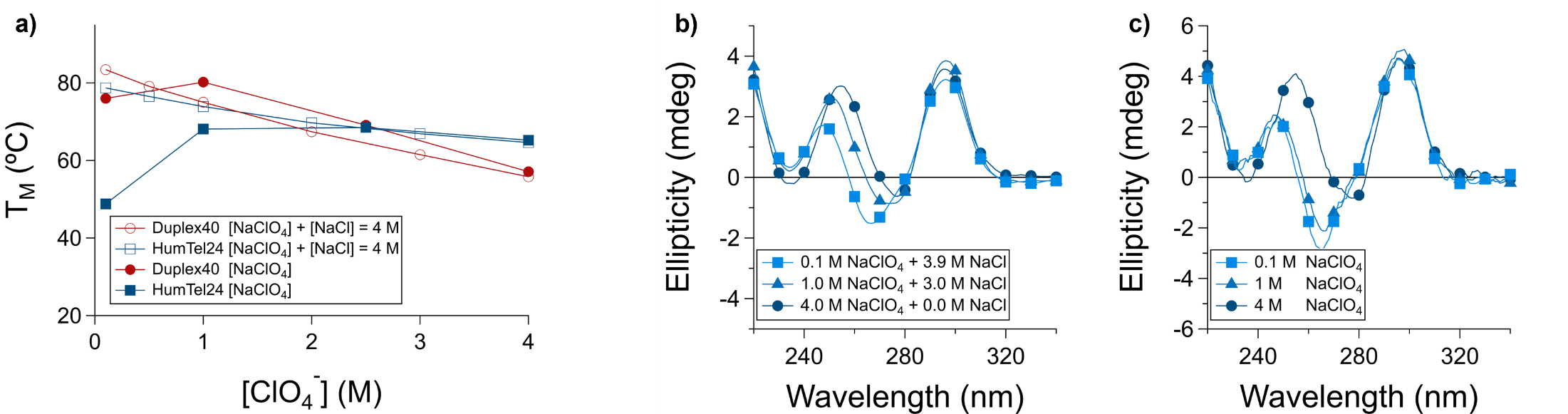


**Supplementary Figure 3.** CD spectra of **HumTel** in varying concentrations of (a) urea and (b) GuCl.
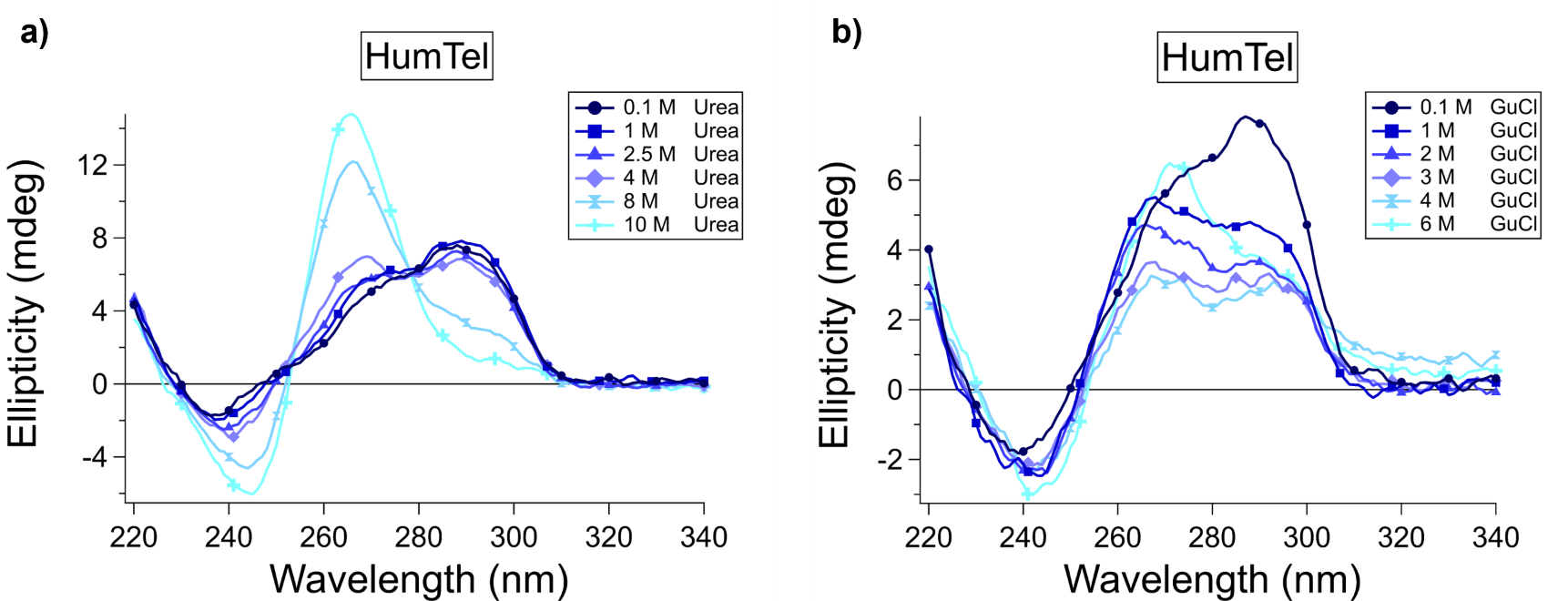


**Supplementary Figure 4.** (a) Melting temperature versus sodium perchlorate and (b) CD spectra of **AGRO100**.
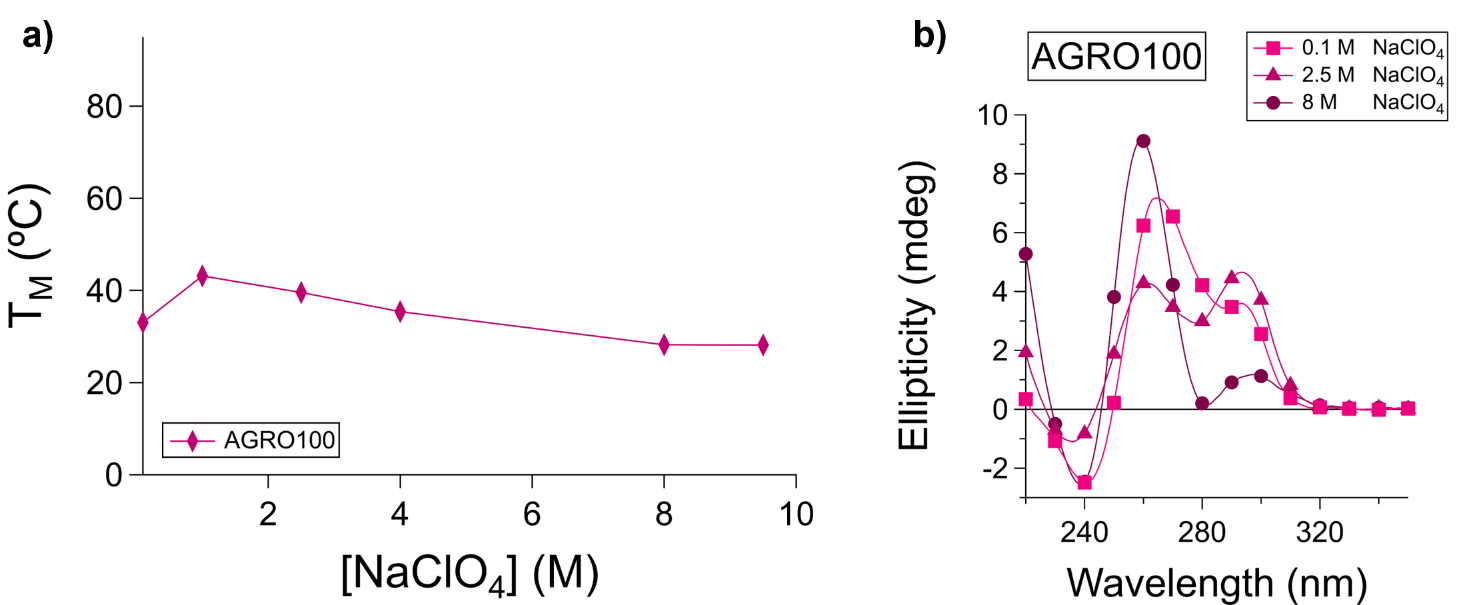


**Supplementary Figure 5.** CD spectra of **HumTel** in varying concentrations of (a) NaSCN and (b) NaI.
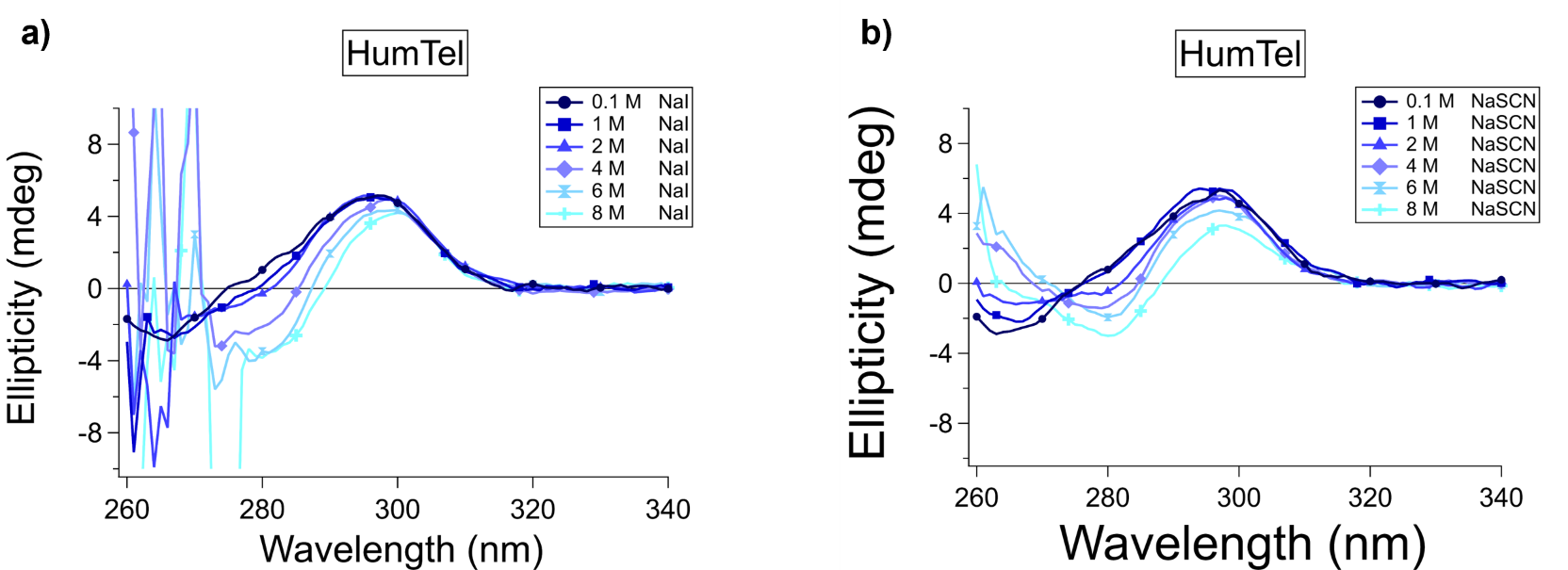


**Supplementary Figure 6.** (a) Melting temperature of **HumTel+LongLoop** compared to **HumTel** in [NaClO_4_]. CD spectra of **HumTel+LongLoop** in varying concentrations of (b) urea, (c) equimolar urea/NaCl, (d) GuCl, and (e) equimolar GuCl/NaCl.
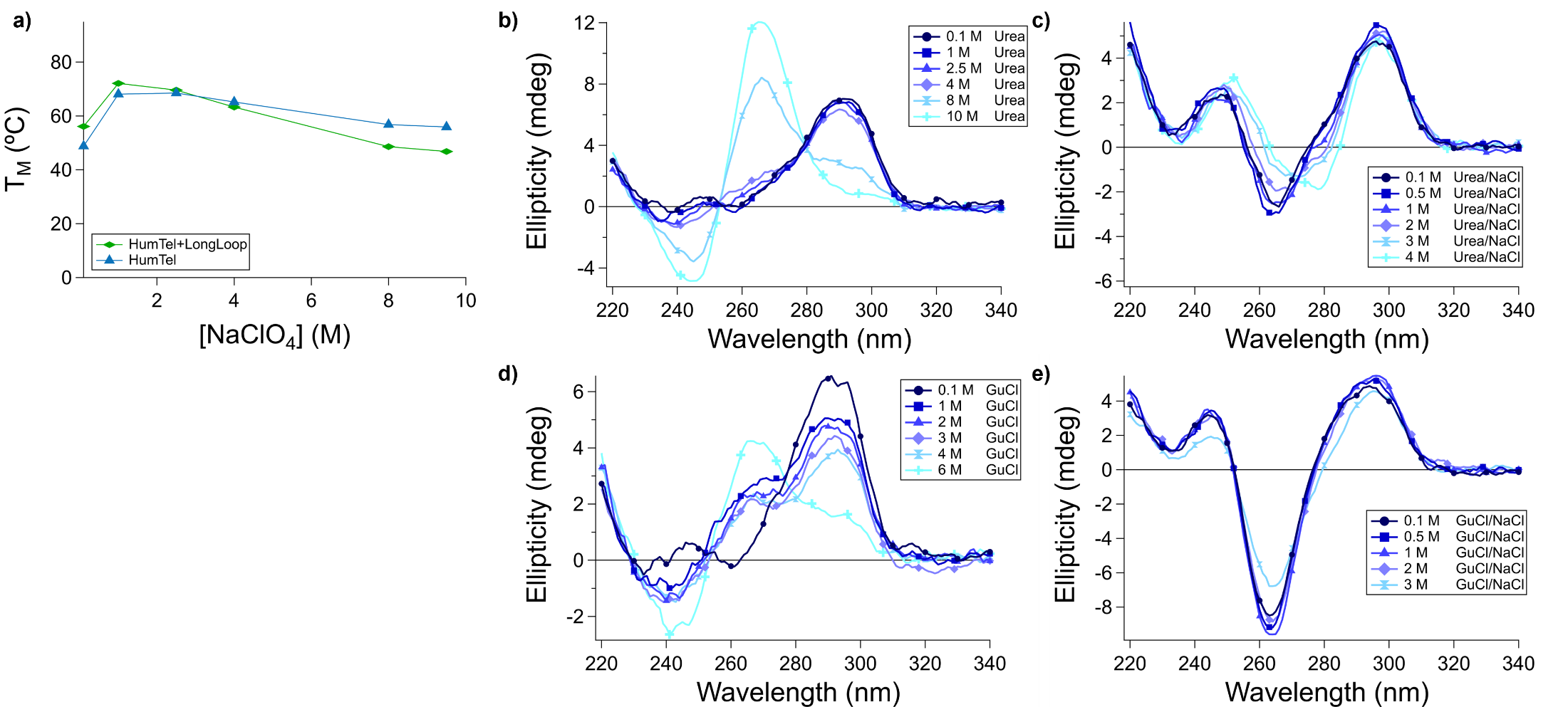


**Supplementary Table 1.** Melting temperatures of nucleic acid structures in varying concentration of NaClO_4_. With standard deviation.

| **Name** | **T_M_  (°C)** | | | | | |
| --- | --- | --- | --- | --- | --- | --- |
|  | **0.1 M NaClO_4_** | **1 M NaClO_4_** | **2.5 M NaClO_4_** | **4 M NaClO_4_** | **8 M NaClO_4_** | **9.5 M NaClO_4_** |
| **Duplex** | | | | | | |
| **Duplex40** | 76.0 ±0.4 | 80.2 ±0.1 | 69.1 ±0.5 | 57.1 ±0.3 | 29.9 ±0.2 | 24.6 ±0.1 |
| **G-Quadruplex** | | | | | | |
| **HumTel** | 48.8 ±0.5 | 68.1 ±0.3 | 68.5 ±0.4 | 65.2 ±0.2 | 56.8 ±0.3 | 55.9 ±0.5 |
| **HumTel-Tetrad** | 31.0 ±0.3 | 45.3 ±0.1 | 42.8 ±0.4 | 39.3 ±0.7 | 25.5 ±0.2 | low |
| **HumTel+Tetrad** | 67.2 ±0.3 | 86.3 ±0.1 | 85.9 ±0.2 | 79.3 ±0.3 | 66.7 ±0.2 | 65.1 ±0.7 |
| **TBA** | low | 28.7 ±0.3 | 29.6 ±0.1 | low | low | low |
| **TBA+Tetrad** | 58.5 ±0.3 | 68.7 ±0.4 | 65.7 ±0.3 | 60.4 ±0.2 | 46.0 ±0.3 | 42.6 ±0.8 |
| **TBA+2Tetrad** | 70.3 ±0.2 | 87.7 ±0.1 | 88.6 ±0.1 | 84.3 ±0.3 | 69.1 ±0.1 | 66.2 ±0.2 |
| **HumTel+LongLoop** | 56.1 ±0.5 | 72.1 ±0.3 | 69.6 ±0.4 | 63.3 ±0.3 | 48.6 ±0.4 | 46.8 ±0.4 |
| **AGRO100** | 33.1 ±0.3 | 43.2 ±0.2 | 39.6 ±0.2 | 35.4 ±0.4 | 28.2 ±0.4 | 28.2 ±1.0 |
| **i-Motif** | | | | | | |
| **cMyc22** | 63.2 ±1.2 | 59.9 ±0.8 | 52.5 ±1.5 | 47.8 ±1.2 | 29.0 ±2.6 | 27.4 ±2.8 |
| **iMotif19** | 69.5 ±1.7 | 67.2 ±1.5 | 62.2 ±1.6 | 56.6 ±1.9 | 39.0 ±2.1 | 34.3 ±2.2 |
| **Py27** | 60.8 ±2.8 | 59.3 ±2.2 | 54.1 ±2.2 | 47.8 ±2.6 | low | low |

**Supplementary Table 2.** Melting temperatures of nucleic acid structures in varying concentrations of ClO­­_4_^-^. Sodium was kept constant using NaCl. With standard deviation.

| **Name** | **T_M_  (°C)** | | | | | |
| --- | --- | --- | --- | --- | --- | --- |
|  | **4 M Na^+^**  **0.1 M NaClO_4_** | **4 M Na^+^**  **0.5 M NaClO_4_** | **4 M Na^+^**  **1 M NaClO_4_** | **4 M Na^+^**  **2 M NaClO_4_** | **4 M Na^+^**  **3 M NaClO_4_** | **4 M Na^+^**  **4 M NaClO_4_** |
| **Duplex** | | | | | | |
| **Duplex40** | 83.4 ±0.3 | 79.1 ±0.3 | 75.0 ±0.2 | 67.4 ±0.6 | 61.5 ±0.4 | 55.8 ±0.5 |
| **G-Quadruplex** | | | | | | |
| **HumTel** | 78.7 ±0.3 | 76.5 ±0.3 | 73.9 ±0.3 | 69.7 ±0.2 | 66.9 ±0.3 | 64.6 ±0.3 |

**Supplementary Table 3.** Melting temperatures of nucleic acid structures in varying concentrations of KCl/urea. Error shown is standard deviation.

| **Name** | **T_M_  (°C)** | | | | | |
| --- | --- | --- | --- | --- | --- | --- |
|  | **100mM KCl**  **0.1 M Urea** | **100mM KCl**  **1 M Urea** | **100mM KCl**  **2.5 M Urea** | **100mM KCl**  **4 M Urea** | **100mM KCl**  **8 M Urea** | **100mM KCl**  **10 M Urea** |
| **Duplex** | | | | | | |
| **Duplex40** | 75.4 ±0.2 | 74.5 ±0.5 | 69.8 ±0.4 | 65.7 ±0.6 | 53.0 ±0.8 | 47.7 ±1.0 |
| **G-Quadruplex** | | | | | | |
| **HumTel** | 60.7 ±0.1 | 56.8 ±0.6 | 49.7 ±0.9 | 42.5 ±1.0 | low | low |
| **HumTel+LongLoop** | 59.2 ±0.5 | 55.6 ±0.6 | 50.1 ±0.6 | 45.5 ±0.5 | 40.7 ±1.3 | 42.9 ±2.2 |

**Supplementary Table 4.** Melting temperatures of nucleic acid structures in varying concentrations of equimolar NaCl/urea. Error shown is standard deviation.

| **Name** | **T_M_  (°C)** | | | | | |
| --- | --- | --- | --- | --- | --- | --- |
|  | **0.1 M NaCl/urea** | **0.5 M NaCl/urea** | **1 M NaCl/urea** | **2 M NaCl/urea** | **3 M NaCl/urea** | **4 M NaCl/urea** |
| **Duplex** | | | | | | |
| **Duplex40** | 71.2 ±0.1 | 79.0 ±0.3 | 83.3 ±0.5 | 80.5 ±0.7 | 74.2 ±1.5 | 67.6 ±1.6 |
| **G-Quadruplex** | | | | | | |
| **HumTel** | 49.4 ±0.4 | 64.9 ±0.3 | 68.5 ±0.3 | 70.2 ±0.7 | 68.0 ±1.4 | 63.6 ±2.1 |
| **HumTel+LongLoop** | 56.2 ±0.1 | 69.1 ±0.4 | 72.6 ±0.1 | 73.1 ±0.7 | 70.5 ±0.9 | 66.1 ±1.3 |

**Supplementary Table 5.** Melting temperatures of nucleic acid structures in varying concentrations of KCl/GuCl. Error shown is standard deviation.

| **Name** | **T_M_  (°C)** | | | | | |
| --- | --- | --- | --- | --- | --- | --- |
|  | **100mM KCl**  **0.1 M GuCl** | **100mM KCl**  **1 M GuCl** | **100mM KCl**  **2 M GuCl** | **100mM KCl**  **3 M GuCl** | **100mM KCl**  **4 M GuCl** | **100mM KCl**  **6 M GuCl** |
| **Duplex** | | | | | | |
| **Duplex40** | 81.1 ±0.6 | 81.1 ±0.1 | 76.9 ±0.1 | 72.7 ±0.1 | 68.7 ±0.3 | 60.1 ±0.3 |
| **G-Quadruplex** | | | | | | |
| **HumTel** | 64.3 ±0.2 | 56.7 ±0.1 | 48.1 ±0.1 | 41.7 ±0.1 | 37.0 ±0.1 | 31.4 ±1.0 |

**Supplementary Table 6.** Melting temperatures of nucleic acid structures in varying concentrations of NaCl/GuCl. Error shown is standard deviation.

| **Name** | **T_M_  (°C)** | | | | |
| --- | --- | --- | --- | --- | --- |
|  | **0.1 M NaCl/GuCl** | **0.5 M NaCl/GuCl** | **1 M NaCl/GuCl** | **2 M NaCl/GuCl** | **3 M NaCl/GuCl** |
| **Duplex** | | | | | |
| **Duplex40** | 81.8 ±0.5 | 84.1 ±0.4 | 81.1 ±0.4 | 74.0 ±0.5 | 63.8 ±1.0 |
| **G-Quadruplex** | | | | | |
| **HumTel** | 51.4 ±0.3 | 60.5 ±0.2 | 61.0 ±0.3 | 56.7 ±0.9 | 48.9 ±0.7 |

**Supplementary Table 7.** Concentration-dependent melting temperatures of G-quadruplexes. Error shown is standard deviation.

| **Name** | **T_M_  (°C)** | | | | | |
| --- | --- | --- | --- | --- | --- | --- |
|  | **0.1 M NaClO_4_** | | | **8 M NaClO_4_** | | |
|  | **1 μM Oligo** | **5 μM Oligo** | **20 μM Oligo** | **1 μM Oligo** | **5 μM Oligo** | **20 μM Oligo** |
| **HumTel** | 48.6 ±0.5 | 48.8 ±0.5 | 48.8 ±0.1 | 57.8 ±1.1 | 56.8 ±0.3 | 57.5±0.1 |
| **HumTel-Tetrad** | 30.4±0.4 | 31.0 ±0.3 | 31.2±0.2 | Low | Low | Low |
| **HumTel+Tetrad** | 65.2±1.0 | 67.2 ±0.3 | 65.1±0.9 | 67.2±0.9 | 66.7 ±0.2 | 64.9±0.4 |
| **TBA** | Low | Low | Low | Low | Low | low |
| **TBA+Tetrad** | 57.4±0.7 | 58.5 ±0.3 | 58.8±0.2 | 47.2±0.2 | 46.0 ±0.3 | 47.6±0.2 |
| **TBA+2Tetrad** | 70.2±0.3 | 70.3 ±0.2 | 70.4±0.2 | 69.2±0.2 | 69.1 ±0.1 | 69.2±0.1 |
| **HumTel+LongLoop** | 55.7±0.4 | 56.1 ±0.5 | 55.5±0.2 | 48.5±0.9 | 48.6 ±0.4 | 49.2±0.1 |
| **AGRO100** | 29.5±1.2 | 33.1 ±0.3 | 31.1±0.3 | 25.8±1.2 | 28.2 ±0.4 | 29.6±1.7 |

**Supplementary Table 8.** Melting temperatures of nucleic acid structures in varying concentrations of NaSCN. Error shown is standard deviation.

| **Name** | **T_M_  (°C)** | | | | | |
| --- | --- | --- | --- | --- | --- | --- |
|  | **0.1 M NaSCN** | **1 M NaSCN** | **2 M NaSCN** | **4 M NaSCN** | **6 M NaSCN** | **8 M NaSCN** |
| **Duplex** | | | | | | |
| **Duplex40** | 76.1 ±0.3 | 81.2 ±0.6 | 73.0 ±0.9 | 52.8 ±0.6 | 29.3 ±0.1 | Low |
| **G-Quadruplex** | | | | | | |
| **HumTel** | 49.5 ±1.0 | 68.8 ±0.5 | 68.4 ±0.3 | 60.1 ±1.0 | 48.9 ±0.3 | 34.6 ±0.8 |
| **HumTel+LongLoop** | 56.6 ±0.4 | 73.2 ±0.1 | 72.0 ±0.2 | 59.0 ±1.5 | 43.1 ±0.2 | Low |

**Supplementary Table 9.** Melting temperatures of nucleic acid structures in varying concentrations of NaI. Error shown is standard deviation.

| **Name** | **T_M_  (°C)** | | | | | |
| --- | --- | --- | --- | --- | --- | --- |
|  | **0.1 M NaI** | **1 M NaI** | **2 M NaI** | **4 M NaI** | **6 M NaI** | **8 M NaI** |
| **Duplex** | | | | | | |
| **Duplex40** | 76.0 ±0.4 | 83.4 ±0.8 | 78.2 ±0.3 | 59.9 ±1.0 | 36.9 ±0.9 | Low |
| **G-Quadruplex** | | | | | | |
| **HumTel** | 48.5 ±0.4 | 71.2 ±0.7 | 73.1 ±0.1 | 66.3 ±0.8 | 52.2 ±1.0 | 34.3 ±1.7 |
| **HumTel+LongLoop** | 55.6 ±0.4 | 75.3 ±0.3 | 75.8 ±0.1 | 64.8 ±0.8 | 45.0 ±0.5 | Low |

**Supplementary Table 10.** Melting temperatures of **Duplex40** derived from A_260_ and A_280_.

|  | **T_M_  (°C)** | |
| --- | --- | --- |
| **[NaSCN]** | **A_260_** | **A_280_** |
| 0.1 | 76.2 | 76.1 |
| 1 | 81.2 | 81.2 |
| 2 | - | 73.0 |
| 4 | - | 52.8 |
| 6 | - | 29.3 |
| 8 | - | Low |
